# Insights on Novel Effectors and Characterization of Metacaspase (RS107_6) as a Potential Cell Death-Inducing Protein in *Rhizoctonia solani*

**DOI:** 10.3390/microorganisms11040920

**Published:** 2023-04-01

**Authors:** N. Kavya, M. K. Prasannakumar, Gopal Venkateshbabu, Vidya Niranjan, Akshay Uttarkar, P. Buela Parivallal, Sahana N. Banakar, H. B. Mahesh, Pramesh Devanna, K. G. Manasa, Tagginahalli N. Shivakumara

**Affiliations:** 1 PathoGenomics Laboratory, Department of Plant Pathology, Gandhi Krishi Vignana Kendra (GKVK), University of Agricultural Sciences, Bangalore 560065, Karnataka, India; kavyan6049@gmail.com (N.K.); gopalvenkateshbabu@gmail.com (G.V.); buella08@gmail.com (P.B.P.); sahananbanakar6391@gmail.com (S.N.B.); manasakg16@gmail.com (K.G.M.); shivutn.bio@gmail.com (T.N.S.); 2 Department of Biotechnology, RV College of Engineering, Bangalore 560059, Karnataka, India; vidya.n@rvce.edu.in (V.N.); akshayuttarkar@gmail.com (A.U.); 3 Department of Genetics and Plant Breeding, Gandhi Krishi Vignana Kendra (GKVK), University of Agricultural Sciences, Bangalore 560065, Karnataka, India; maheshhbg@gmail.com; 4 Rice Pathology Laboratory, All India Coordinated Rice Improvement Programme, Gangavathi, University of Agricultural Sciences, Raichur 584104, Karnataka, India; parmi.iari@gmail.com

**Keywords:** *Rhizoctonia solani*, necrotrophs, cell death effectors, metacaspase, host–pathogen interaction

## Abstract

Effectors play an important role in host–pathogen interactions. Though an economically significant disease in rice, knowledge regarding the infection strategy of *Rhizoctonia solani* is obscure. In this study, we performed a genome-wide identification of the effectors in *R. solani* based on the characteristics of previously reported effector proteins. A total of seven novel effectors (designated as RS107_1 to RS107_7) in the disease mechanism of *R. solani* were identified and were predicted to be non-classically secreted proteins with functionally conserved domains. The function, reactivity, and stability of these proteins were evaluated through physiochemical characterization. The target proteins involved in the regulation of rice defense mechanisms were identified. Furthermore, the effector genes were cloned and RS107_6 (metacaspase) was heterologously expressed in *Escherichia coli* to obtain a purified protein of ~36.5 kDa. The MALD-TOF characterization confirmed that the protein belonged to a metacaspase of the Peptidase_C14 protein family, 906 bp in size, and encoded a polypeptide of 301 amino acids. These findings suggest that the identified effectors can potentially serve as a virulence factor and can be targeted for the management of sheath blight in rice.

## 1. Introduction

The basidiomycete *Rhizoctonia solani* is a necrotrophic pathogen with a broad host range that infects food, fiber, and horticultural crops worldwide. It causes seed decay, seedling damping-off, root rot, black scurf, sheath blight, and stem canker in a diversity of crops [1]. Sheath blight disease, caused by isolates belonging to the anastomosis group AG1-IA, is an economically important fungal disease in rice. The pathogen can spread rapidly under favorable conditions, causing up to a 50% loss in rice yields. As rice is considered a staple food for half of the global population, food security is directly affected by the rice sheath blight disease [2]. By virtue of the variability, broad host range, and polygenic nature of the resistance trait, breeding for sheath blight-resistant varieties is complicated. Traditional control measures such as commercial fungicides and cultural practices have limited efficacy, and can have a negative environmental impact [3]. Therefore, understanding the molecular interplay between *R. solani* and its host rice will aid in devising novel crop protection measures.

Necrotrophs have long been perceived as simple pathogens that employ phytotoxins and cell wall-degrading enzymes to invade their hosts and acquire nutrients; however, the perspective towards the pathogenic mechanism in necrotrophic pathogens has shifted in the last decade. The availability of draft genome sequences for different anastomosis groups of *R. solani* (AG1-IA, AG1-IB, AG3, AG8, and AG2-2IIIB) has provided a wealth of genomic information that can be used to comprehend its pathogenic mechanism [4,5,6,7,8]. The *R. solani* genome encodes small, secreted proteins called effectors, which are pathogen-encoded proteins that manipulate the host cell physiology and target the key components of host metabolism, transcription, and signal transduction to facilitate infection and disease development [9]. The effectors in necrotrophic phytopathogenic fungi can have various roles, such as altering host cell structure, inducing necrosis, protecting hyphae from degradation, triggering a defense response, or avoiding recognition by the host [10]. Several cell death-inducing effectors have been identified in *R. solani*, such as a thaumatin-like protein (RsAG8_08836), polygalacturonase genes (RsPG3 and RsPG4), a lipase domain-containing effector (AGLIP1), RsIA_NP8, and RsMf8HN, which were found to induce tissue necrosis in *Nicotiana benthamiana* leaves upon infiltration, signifying their possible roles as virulence factors [11,12,13,14,15].

In addition to causing necrosis at a later stage of infection, *R. solani* uses effectors at an early stage of infection to suppress the host immunity. For example, LysM effectors contain lysin motif domains that bind to chitin, a major component of the fungal cell wall, and interferes with chitin recognition by the plant immune system. RsLysM is one such effector identified in *R. solani* [16]. Another mechanism involves using small, secreted proteins, such as RsCRP1, to impair ROS production in the mitochondria and chloroplasts, thereby suppressing defense responses [17]. The RsRlpA (rare lipoprotein A) effector has been shown to suppress the oxidative burst and hypersensitive response induced by the PAMP-triggered immunity (PTI) response [18]. Therefore, identifying the effector genes in the *R. solani* genome can assist researchers in understanding how the pathogen interacts with its host and identify potential targets for developing new antifungal strategies.

In the present study, bioinformatics tools were used to identify novel cell death effectors in the genome of the *R. solani* isolate RS24, and determine their expression patterns during the infection stage. Furthermore, the interaction of the putative effectors with the rice proteins involved in the programmed cell death mechanism was also investigated, implying a key role played by the predicted proteins in the manipulation of host immunity. Among the seven effectors, the Peptidase_C14 metacaspase protein (RS107_6) was expressed and characterized using MALDI-TOF analysis. Overall, this research study was aimed at identifying the potential effector proteins and provided new perspectives for understanding the molecular mechanisms in *R. solani* pathogenesis.

## 2. Material and Methods

### 2.1. Fungal Strain, Plant Material, and Rice Inoculation

The phytopathogenic fungus *Rhizoctonia solani* isolate RS107 was collected from rice fields infected with sheath blight at V.C. Farm, Mandya, Karnataka, India, and isolated on potato dextrose agar (PDA) medium. The inoculated plates were incubated at 28 ± 1 °C for 5–6 days. The isolated fungal sample was identified based on morphological characteristics [19] and ITS rDNA sequencing [20]. Further, the pure fungal cultures were preserved on PDA slants at 4 °C. The susceptible rice cultivar (IR64) was grown and maintained in a glasshouse at 25 ± 5 °C, with 60% RH, and 16 h light/8 h dark conditions. The plants were inoculated at the maximum tillering stage by placing mycelial disks (8 mm) of the RS107 isolate grown on PDA for 7 days at 28 ± 1 °C, following the method described in Park et al. [21]. Infected sheaths were harvested at 0, 48, and 72-h post-inoculation (hpi) to analyze the gene expression of cell death effectors in rice plants.

### 2.2. In Silico Identification and Characterization of Putative Cell Death Effectors in R. solani

To identify the novel cell death effectors in *R. solani,* we obtained the complete genome data (SUB9199492, unpublished) of the virulent isolate RS24 from the PathoGenomics lab, Department of Plant Pathology, UAS, Bangalore, India, and performed local BLASTp search for the necrotrophic proteins obtained from the PHI-base: pathogen–host interaction database (http://www.phibase.org/; accessed on 20 March 2022). Potential effectors were predicted using the fungal effector predictor EffectorP 3.0 [22] and assessed for their putative function using BLASTKOALA (https://www.kegg.jp/blastkoala/; accessed on 20 March 2022). Conserved domains were inspected using CDART (https://www.ncbi.nlm.nih.gov/; accessed on 10 April 2022). The peptide for secretion signals was predicted using the SignalP4.1 (http://www.cbs.dtu.dk/services/SignalP/; accessed on 10 April 2022) server, and non-classically secreted proteins lacking signal peptides and cleavage sites were predicted using OutCyte (http://www.outcyte.com/; accessed on 18 April 2022). For transmembrane domain and glycophosphatidylinositol (GPI) anchor motif search, TMHMM v2.0 (https://services.healthtech.dtu.dk/services/TMHMM-2.0/; accessed on 25 April 2022 ) and PredGPI (http://gpcr.biocomp.unibo.it/predgpi/index.htm/; accessed on 25 April 2022) were employed, respectively; and sub-cellular localization was predicted using the WoLFPSORT (https://wolfpsort.hgc.jp/; accessed on 25 April 2022) server.

Physiochemical parameters in proteins can delineate their functional properties, behavior, and stability under various in vitro conditions. Therefore, Expasy ProtParam (https://web.expasy.org/protparam/; accessed on 14 July 2022) was used to compute various physiochemical properties such as amino acid composition, molecular weight, isoelectric point (pI), negatively and positively charged residues, instability index (II), aliphatic index (AI), and grand average hydropathicity (GRAVY).

### 2.3. Phylogenetic Analysis of Cell Death Effectors

A BLASTp search of amino acid sequences in the identified cell death effectors was performed for the non-redundant NCBI protein database (default parameters) to find homologues among pathogenic fungi. The homologous sequences from various fungal species were retrieved from NCBI and the amino acid sequences were aligned using ClustalW [23]. The phylogenetic tree was constructed using the neighbor-joining tree method with a bootstrap test of 1000 replicates using MEGA11 [24].

### 2.4. Molecular Docking Analysis and Molecular Dynamic (MD) Simulation Studies

The three-dimensional structures of the effector proteins were predicted using the template-based protein structure modeler RaptorX [25] because of the low structural identity with the protein structures available in the Protein Data Bank (PDB). Similarly, the 3D structures of rice genes involved in defense mechanisms, including programmed cell death, extracted from RPGdb, were also predicted. The accuracy of the developed models was validated by computing the Ramachandran (RC) plot [26] using the PROCHECK program included in the SAVESv6.0 server pipeline (https://saves.mbi.ucla.edu/; accessed on 17 September 2022).

Molecular docking was initiated using Hex v8.0.0 docking software [27] to study the molecular interactions between the rice target proteins and *R. solani* cell death effectors. Shape-only correlation and 3D Fast Lite as an FFT mode with a grid size of 0.6, receptor range of 180, ligand range of 180, twist range of 360, and distance range of 40 were the parameters used for the docking procedure. The docked structures were visualized and analyzed using PyMol v2.5.4. To assess the conformational stability of the interaction, MD simulations of the RS107_6-LOC_Os03g06410 complex were performed for a duration of 100 ns using the Schrodinger Desmond 2021–2022 package [28]. The OPLS_2005 force field [29] and orthorhombic TIP3P water model [30] were used in the simulations. Na+ ions were used to neutralize the solvated system. The MD simulations for the energy-minimized system were conducted under an NVT ensemble, maintaining the temperature and pressure at 310 K and 1 atm, respectively. The trajectories recorded during the simulation were analyzed using the Simulation Interaction Diagram Wizard.

### 2.5. RNA Extraction, cDNA Synthesis and Cloning of R. solani Genes Encoding Putative Cell Death Effectors

Total RNA from the fungal mycelia (100 mg) grown in potato dextrose broth (PDB) medium was extracted using the NucleoSpin^®^ RNA Plus kit (Macherey-Nagel, Düren, Germany) according to the manufacturer’s instructions. First-strand cDNA was synthesized using the QuantiTect^®^ Reverse Transcription Kit (QIAGEN, Hilden, Germany) following the manufacturer’s protocol and was used for further studies. PCR primers for cell death effectors were designed and listed in Table S1. The complete coding sequence (CDS) of each effector was PCR-amplified from 50 ng cDNA (RS107 isolate) and the reaction mixture for each gene consisted of 5 μL of 2X master mix (TaKaRa, Naha, Japan), 10 pmol each of forward and reverse primers, and sterile distilled water in a final volume of 10 μL. The thermal cycler was programmed for initial denaturation at 94 °C for 4 min, 35 cycles at 94 °C for 1 min, 60 °C for 1 min, and 72 °C for 1 min, and a final extension at 72 °C for 10 min. The PCR products were purified using the NucleoSpin^®^ Gel and PCR Clean-up kit (Macherey-Nagel, Düren, Germany) and cloned into pMD20-T vector using a Mighty TA Cloning kit (TaKaRa Bio, Shiga, Japan), followed by transformation of competent *E. coli* DH5α cells (Invitrogen, Waltham, MA, USA). Transformants were confirmed using colony PCR and sequencing.

### 2.6. Expression and Purification of Recombinant Protein RS107_6

RS107_6 was PCR amplified using high-fidelity polymerase (QIAGEN, Hilden, Germany) with specific primers containing *BamHI* and *HindIII*. Further, the PCR amplified RS107_6 gene and pET-28a(+) were double-digested with *BamHI* and *HindIII,* followed by ligation and transformation into the *E. coli* BL21 (DE3) strain (Invitrogen, Waltham, MA, USA). The orientation of the target gene in the recombinant plasmid pET-28 a(+)/RS107_6 was confirmed by sequencing using T7 primers and SnapGene version 6.2. The expression of the recombinant protein was induced with isopropyl β-1-D-thiogalactopyranoside (IPTG; 0.1 mM) overnight at 16 °C. The expressed protein was purified using Ni-NTA affinity chromatography (QIAGEN, Hilden, Germany). The histidine-tagged protein bound to the matrix was eluted and dialyzed with 1X Tris buffer, pH 7.5 (50mM Tris-HCl) for 24 h at 4 °C. Total protein concentration was estimated using the Bradford method [31]. The expression and purity of the recombinant protein were analyzed using SDS-PAGE, followed by staining with Coomassie Brilliant Blue R-250.

### 2.7. MS Analysis of Recombinant RS107_6 Protein Using TripleTOF 5600+

The band of the heterologously expressed recombinant protein (RS107_6) was excised from the CBB-stained SDS-PAGE gel, and in-gel digestion of proteins was performed. The dried peptide mixture was re-dissolved in 0.1% formic acid and 3% ACN in water supplemented with indexed retention time (iRT) peptide standards according to the manual (Biognosys, Schlieren, Switzerland). The samples were introduced into the TripleTOF MS 5600+ through a Nanospray III source (Sciex, Framingham, MA, USA) with an electrospray potential of 2.2 kV. Mass spectrometry was performed using information-dependent acquisition (IDA). The MS spectra were searched in the NCBI_*Rhizoctonia solani* database using the MASCOT search engine (version 2.4).

### 2.8. Gene Expression Analysis (qPCR) of Predicted Effector Genes in Planta

For in planta expression, total RNA from the infected rice sheaths (100 mg) was harvested at 0, 48, and 72-hpi and was extracted using the method described in Section 2.5. Specific primers for *q*PCR were designed (Table S2) and 18S rRNA was used as an endogenous control for normalization. To analyze the relative expression of cell death effectors during infection, amplification was performed using SYBR Green (TaKaRa, Japan) in CFX96 *q*RT-PCR (Bio-Rad, Hercules, CA, USA), and relative expression was calculated using the 2^−ΔΔCt^ method [32].

### 2.9. Statistical Analysis

In this study, all of the experiments were performed in triplicate. The data obtained were expressed as the mean ± standard error and statistically analyzed using GraphPad Prism software version 9.5.1.733. One-way analysis of variance (ANOVA) was performed using Fisher’s LSD at *p* < 0.05, to determine the significance of differences between the means of the control and treatment.

## 3. Results

### 3.1. Molecular Identification of Fungal Isolate

The molecular identification of the fungus was carried out based on ITS rDNA sequencing, and BLASTn analysis revealed that the sheath blight in rice was caused by *Rhizoctonia solani.* The ITS sequence of *Rhizoctonia solani* (GenBank ID: MK788181) isolated in our study showed a 99.6% similarity to the *R. solani* isolate JK-2016-14 and belonged to the anastomosis group AG1-IA clad (Figure S1).

### 3.2. Genome-Wide Identification of Putative Cell Death Effectors in R. solani

**Figure 1:**
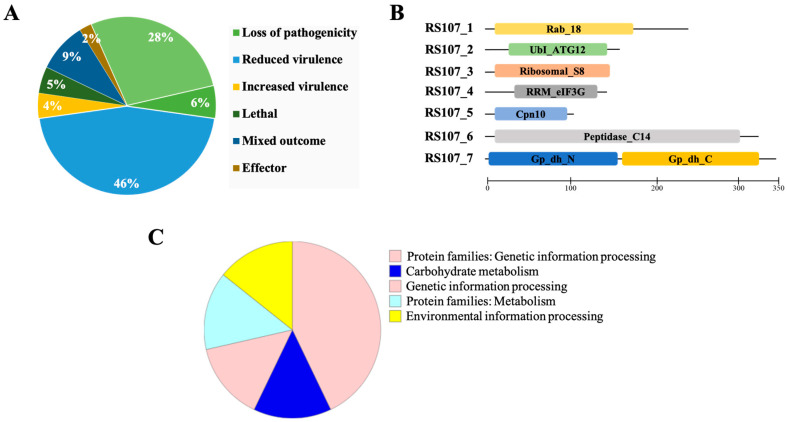
Characterization of cell death effectors (RS107_1 to RS107_7) (**A**) Pathogenicity-associated genes in *R. solani* identified through PHI-base analysis. (**B**) Predicted conserved domains in seven cell death effectors. (**C**) Functional annotation and categorization of cell death effectors using BLASTKOALA.

A genome-wide search for cell death effectors in the *R. solani* isolate RS24 (GenBank submission no. SUB9199492, unpublished) was performed using the effectors of necrotrophic phytopathogens as a query. The proteins homologous to necrotrophic proteins catalogued in the PHI database were identified and found to be 685 in number. The highest proportion (493) was attributed to pathogenicity and virulence, followed by the group of unaffected pathogenicity proteins (192). The majority of these 493 proteins (312) were partially involved in determining pathogenicity (reduced virulence), about 40 proteins were primary determinants of pathogenicity, 33 proteins were lethal, 31 proteins were involved in the negative regulation of virulence (increased virulence), and 15 were identified as effectors. Approximately 62 proteins were found to have mixed-outcome phenotypes. A detailed description of the various pathogenicity factors identified in this study is presented in Figure 1A. Of the identified pathogenicity genes, 57 were verified with the EffectorP standards for effector prediction. Seven effector proteins (designated RS107_1 to RS107_7) were selected from among 57 effectors for the current investigation, as they were uncharacterized in *R. solani*.

### 3.3. In Silico Characterization of Putative Effector Proteins

The seven novel cell death effectors were predicted to be non-classically secreted proteins with no signal peptides, transmembrane domains, or GPI-anchor sites. The length of these proteins ranged from 98 (RS107_5) to 343 (RS107_7) amino acids with at least one cysteine residue (except for effector RS107_4, which had no cysteine residues). The localization of a protein was related to its function within the host plant, and the investigations into the subcellular localization of the cell death effector proteins revealed that the majority of the effectors were cytoplasmic, while effector RS107_3 was either cytoplasmic or apoplastic, which was predicted using the WoLF PSORT protein localization predictor (https://wolfpsort.hgc.jp//; accessed on 25 April 2022). The seven cell death proteins contained conserved domains (Figure 1B), including the Rab_18 domain (cd01863), ubiquitin-like autophagy protein ATG12 (PF04110), ribosomal_S8 (PF00410), eIF-3G RNA recognition motif (cd12408), chaperonin Cpn10 (cd00320), peptidase_C14 metacaspase domain (PF00656), glyceraldehyde-3-phosphate dehydrogenase with N-terminal NAD binding (PF00044), and C-terminal catalytic domain (PF02800). The biological roles of the putative effectors were analyzed and categorized based on the KO system, as shown in Figure 1C, including protein families: genetic information processing (three entries), protein families: metabolism (one entry), environmental information processing (one entry), carbohydrate metabolism (one entry), and genetic information processing (one entry).

### 3.4. Physiochemical Characterization

The physicochemical properties of the effector proteins were computed using the ProtParam tool and were tabulated in Table 1. Effectors RS107_1, RS107_4, RS107_6, and RS107_7 were acidic (pI 5.39–6.6), while the others were alkaline (pI 9.23–10.33). Except for RS107_6, which had an instability index exceeding 40, all of the proteins were stable in nature. Cut off values of <40 and >40 were used to identify the stable and unstable proteins; a high aliphatic index (49.92–113.77) indicated the thermal stability of the effectors over a wide temperature range [33,34]. Except for RS107_5, the proteins were hydrophilic with a negative GRAVY index [35].

### 3.5. Cell Death Effectors Are Highly Conserved among R. solani Isolates

**Figure 2:**
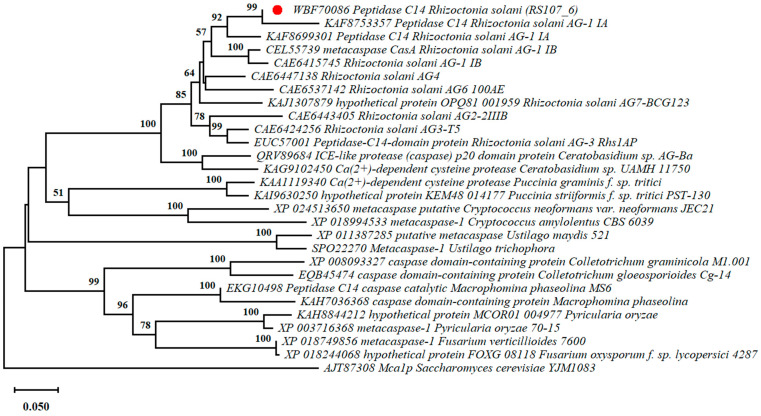
Phylogenetic analysis of aligned amino acid sequences in RS107_6 with its reference protein sequences using the neighbor-joining algorithm in MEGA11.

To analyze the conserved degree of the homologs of the identified novel effectors in the fungi, a BLASTp search using the non-redundant protein database was performed. The evolutionary relationship of the putative effector proteins among the various fungal species belonging to Ascomycetes and Basidiomycetes was analyzed using a phylogenetic tree and two major clusters formed; one having proteins from Basidiomycetes and the other from Ascomycetes. The effectors exhibited amino acid sequence similarity with orthologs in other fungi such as *Saccharomyces cerevisiae* (29–77%), *Magnaporthe oryzae* (38–78%), *Colletotrichum* sp. (50–65%), *Ustilago maydis* (60–78%), and *Macrophomina phaseolina* (37–67%). However, for all of the seven effectors, a high amino acid sequence similarity (92–99%) was observed among *R. solani* strains belonging to the different anastomosis groups and hence were located in one clad (Figure 2 and Figure S2A–F). The RS107_3, RS107_6, and RS107_7 proteins were observed to be highly conserved among the pathogenic fungi with protein similarity ranging between 65 and 78%.

### 3.6. Modelling of Effectors and Their Interacting Partners in Rice System

The binding affinity of the cell death effectors to the genes involved in the programmed cell death mechanisms, which are crucial for the defense response in rice, was investigated using molecular docking studies. A total of 87 programmed cell death cascade proteins in rice were obtained from the RPGdb database, and three-dimensional models for the effector and rice proteins were developed using RaptorX (Figures S3A and S4A). The 3D structures were further validated by computing an RC plot using PROCHECK software. According to the RC plot statistics, the modelled structures had 83–95% of the amino acid residues in the most favored region, 5.0–12.4% of the residues in the additionally allowed region, 0–4.5% in the generously allowed region, and 0–3% in the disallowed region (Figures S3B and S4B). Over 80% of the amino acid residues were found in the most favored region, indicating a quality protein model [36]. Thus, the predicted models were reliable for exploring the interactions between the pathogen and host proteins using the docking analysis.

### 3.7. Molecular Docking, Simulations, and Analysis of Docked Structures

**Figure 3:**
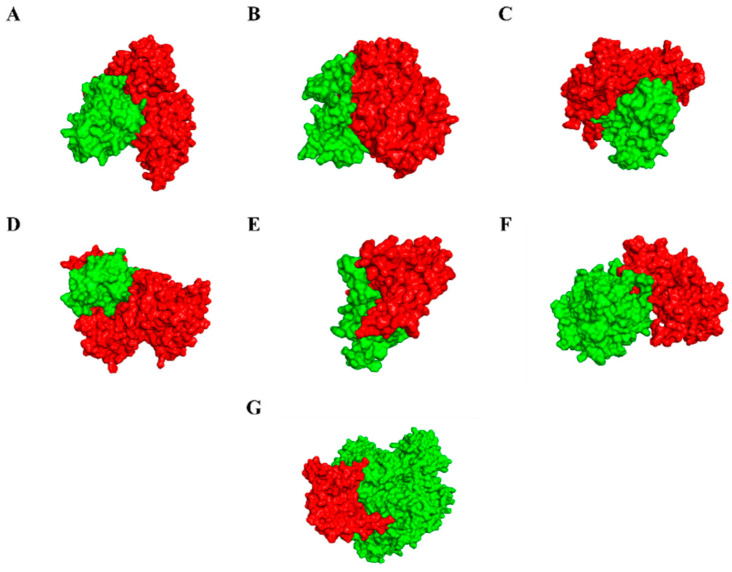
Molecular docking interaction studies of cell death effectors (green) in *R. solani* with the cell death cascade genes (green) in rice. (**A**) RS107_1-LOC_Os04g56480. (**B**) RS107_2-LOC_Os01g56330. (**C**) RS107_3-LOC_Os03g10750. (**D**) RS107_4-LOC_Os01g02720. (**E**) RS107_5-LOC_Os09g38580. (**F**) RS107_6-LOC_Os03g06410. (**G**) RS107_7-LOC_Os02g36974.

**Figure 4:**
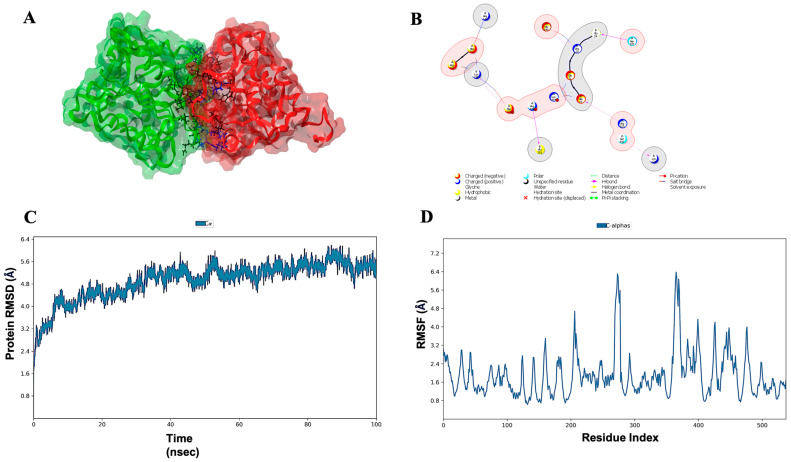
MD simulations studies for the effector–target complex, RS107_6-LOC_Os03g06410 (**A**) and (**B**) Molecular interaction profile of crucial amino acids of effector (RS107_6) with the host protein (LOC_0s03g06410). (**C**) RMSD plot for the pathogen–host protein complex during 100 ns simulation. (**D**) RMSF of Cα atoms of interacting complex along the course of 100 ns simulation.

Eighty-seven programmed cell death cascade proteins from rice were docked with the cell death effectors in *R. solani*. Over ten best hits (rice proteins) were obtained for each effector and eventually, one lead protein was selected. Based on the molecular docking scores, the lead proteins for RS107_1 to RS107_7 were LOC_Os04g56480 (−732.80 kcal/mol), LOC_Os01g56330 (−751.50 kcal/mol), LOC_Os03g10750 (−847.60 kcal/mol), LOC_Os01g02720 (−704.50 kcal/mol), LOC_Os09g38580 (−759.20 kcal/mol), LOC_Os03g06410 (−832.26 kcal/mol), and LOC_Os02g36974 (−210.10 kcal/mol), respectively (Figure 3). The role of these proteins in the defense mechanisms of the rice plants are described in Table 2. Following the docking analysis, a 100 ns MD simulation was performed to investigate the stability of the pathogen–host protein complex RS107_6-LOC_Os03g06410. The interaction profile during the MD simulation for the RS107_6-LOC_Os03g06410 interaction complex are depicted in Figure 4A,B. The interaction between the effector and the rice protein observed through the course of the simulation revealed that strong hydrogen bonding drove the binding of RS107_6 with LOC_Os03g06410. Figure 4B shows that the interaction included residues Glu19-Arg823, Gly24-Asn937, Ala201-Arg778, and Lys242-Ser892 forming hydrogen bonds; Arg142-Asp766 forming salt bridges; and Glu19-Lys819, Glu21-Lys819, Arg23-Glu898, Lys195-Asp766, Lys195-Asp768, and Lys195-Asp772 forming hydrogen bonds and salt bridges. The simulation trajectories revealed that the root mean square deviation (RMSD) values increased steadily from 3.2 to 4.8 Å until 30 ns, gradually attained equilibrium, and remained stable throughout with a maximum RMSD value of 6.2 Å (Figure 4C). The root mean square fluctuation (RMSF) data were useful for analyzing conformational changes along the protein length. Residues between 250–300 and 350–400 were observed to have the highest peaks with an RMSF value of 6.4 Å, indicating that only a small portion of the amino acids were subjected to conformational changes during the simulation (Figure 4D). This demonstrated that RS107_6 formed a very stable complex with the rice protein LOC_Os03g06410.

### 3.8. Cloning, Expression, and Purification of the Recombinant RS107_6 Protein

Complete coding sequences for all seven cell death effectors were generated and submitted to the NCBI database. The GenBank accession IDs obtained for those effectors are listed in Table S3.

**Figure 5:**
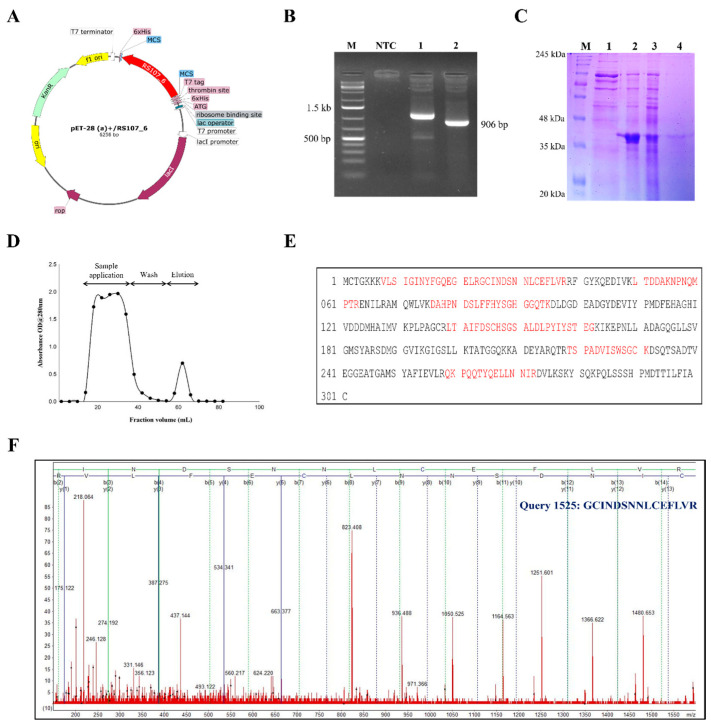
Heterologous expression and characterization of effector protein RS107_6. (**A**) Recombinant pET-28 a(+)/RS107_6 vector construct for the expression of RS107_6 effector. (**B**) Colony PCR confirmation of *E. coli* transformants for pET-28 a(+)/RS107_6 with Lane 1: T7 primers, Lane 2: gene-specific primers, and M: GeneRuler 1kb plus ladder (Thermo Scientific, Waltham, MA, USA). (**C**) SDS-PAGE showing the expression of his-tagged RS107_6 protein. M: pre-stained protein marker (Sigma-Aldrich, St. Louis, MO, USA), Lane 2: uninduced control, Lane 3: pellet, Lane 4: supernatant, Lane 5: purified protein. (**D**) Elution profile of RS107_6 protein on Ni-NTA column chromatography. Crude protein was applied on to Ni-NTA matrix and fractions of 4 mL were collected at the flow rate of 12 mL/h and analyzed for the presence of target protein. (**E**) RS107_6 protein sequence with matching peptides (red) identified using MASCOT search engine. (**F**) MALDI-TOF MS spectra of recombinant protein (RS107_6).

The double-digested fragment of RS107_6 (906 bp) was ligated to the bacterial expression vector pET-28 a(+), and the recombinant construct of pET-28 a(+)/RS107_6 (Figure 5A) was analyzed for protein expression in the *E. coli* BL21 (DE3) cells. The positive *E. coli* cells harboring pET-28 a(+)/RS107_6 (Figure 5B) were induced for protein expression with IPTG (0.1 mM) at 16 °C. A distinctly expressed protein band of ~36.5 kDa was observed in the pellet (Figure 5C); therefore, the inclusion bodies found in the pellet were solubilized to isolate the intact protein fraction. The solubilized inclusion bodies were purified using Ni-NTA affinity column chromatography. The recombinant RS107_6 was eluted using 250 mM of imidazole. Approximately 21 protein fractions (4 mL) were collected, and a single absorption peak was observed between fractions 14 and 18 (Figure 5D). All target protein fractions were pooled, dialyzed with Tris-HCl (pH 7.5), and lyophilized. The expressed protein purified from the crude extract yielded approximately 11.6 mg, representing 41.8% of the total proteins. The purification and protein turnover of his-tagged RS107_6 are summarized in Table 3.

### 3.9. RS107_6 Protein Characterization

The recombinant protein fraction (14-18) purified from the Ni-NTA column displayed a single band in the SDS-PAGE analysis with CBB (Coomassie Brilliant Blue) staining, with a relative molecular weight of ~36.5 kDa. The single protein band was excised for tryptic digestion and further analyzed using MALDI-TOF MS. The identity of the purified recombinant RS107_6 protein was confirmed using matrix-assisted laser desorption ionization time-of-flight (MALDI-TOF) mass spectrometry. The resulting peptide mixture obtained was analyzed using MALDI-TOF MS to produce the MS spectrum. The spectrum/peak lists (*m*/*z* ratio) obtained were subjected to a peptide mass fingerprinting (PMF) search in the Mascot Database (https://www.matrixscience.com/search_form_select.html/; assessed on 10 December 2022). The seven peptides: TSPADVISWSGCK, LTDDAKNPNQMPTR, VLSIGINYFGQQGELR, GCINDSNNLCEFLVR, QKPQQTYQELLNNIR, DAHPNDSLFFHYSGHGGQTK, and LTAIFDSCHSGSALDLPYIYSTEGK matching RS107_6 accounted for 39.20% of the total protein (Figure 5E). The peptide GCINDSNNLCEFLVR showed the highest score of 119, and its corresponding spectra are depicted in Figure 5F. The recombinant protein RS107_6 was identified as metacaspase-1 in the Peptidase_C14B protein family (UniProt ID. A0A8H2XLR2_9AGAM) with cysteine-type peptidase activity during apoptosis. Further, the BLASTp search in the NCBI non-redundant database revealed that the peptide shared high sequence similarity with Peptidase_C14 (KAF8753357.1), the ICE-like protease (p20) domain protein (XP_043178497.1) in *Rhizoctonia solani*. The mass spectrum therefore supported that the purified protein was metacaspase.

### 3.10. Relative Expression Patterns of Effector Genes during Rice Infection

**Figure 6:**
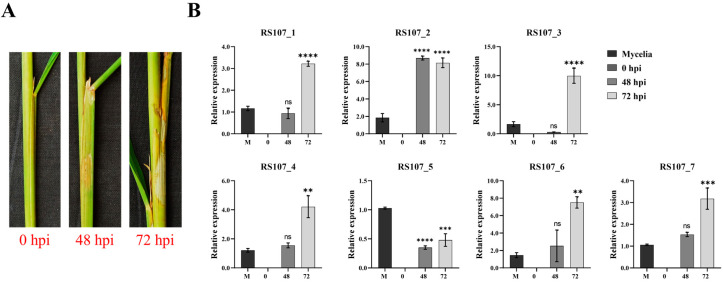
In planta gene expression analysis (*q*PCR) of seven cell death effectors. (**A**) Progression of disease from inoculation to the development of necrotic lesions. (**B**) Relative expression pattern of effectors (RS107_1 to RS107_7) in susceptible rice genotype (IR64) at different time intervals. All of the values are mean ± SE with three biological replicates. Statistics were performed using one-way ANOVA. Asterisk indicates significant difference; ** *p* = 0.0004, *** *p* = 0.0001, **** *p* <0.0001, ns–no significant difference.

The role of cell death effectors in the development of sheath blight disease was explored by determining their expression patterns in planta at 48 and 72 hpi, while the fungal mycelia grown in the PDB medium served as the control. From the data presented in Figure 6B, it was evident that with the exception of the RS107_5 gene, which was downregulated in planta, all of the other six effectors were upregulated during infection, predominantly at 72 hpi (cell death stage, Figure 6A). The relative expression of RS107_1 was not significantly affected at 48 hpi; however, the expression of RS107_1 increased three-fold at 72 hpi in comparison to the control (1.17). A similar trend was observed for the genes RS107_4 and RS107_7, where a four-fold increase in the relative expression was noticeable at 72 hpi relative to the control (1.22). Similarly, the transcript abundance of RS107_6 increased proportionately to the disease progression, which was evident from the two- and seven-fold increases after 48 and 72 hpi relative, respectively to 1.46 (control). While the expression of the RS107_2 gene increased drastically by eight-fold during infection at both 48 and 72 hpi compared to the control (1.86), the expression of the RS107_3 gene reduced by 0.31 at 48 hpi and escalated nine-fold at 72-hpi over the control (1.67). Conversely, for the gene RS107_5, a decrease in the transcript abundance by 0.68 (48 hpi) and 0.55 (72 hpi) times was observed in planta, while the expression was slightly higher in vitro (1.03). Overall, the effectors RS107_2 and RS107_3 were highly upregulated, followed by RS107_1, RS107_4, RS107_6, and RS107_7. 

## 4. Discussion

Effectors play a crucial role in interacting with the host plants and establishing infection. The devastating impact of the necrotrophic fungi, *R. solani*, on rice production has drawn attention to untangle the molecular mechanisms of pathogenesis through effector characterization. However, due to fewer numbers of effectors identified in *R. solani*, knowledge and information regarding its infection strategy are obscure. Fungal effectors lack conserved sequence features akin to oomycetes [46]. Hence, the criteria based on the previously identified effectors including a signal peptide, small size, and cysteine content are employed for effector prediction. However, not all of the proteins failing to comply with these criteria are non-effectors [47]. In this research study, we predicted 685 pathogenicity factors including 57 potential effectors from the genome of virulent isolate RS24, which were mainly involved in determining the severity spectrum of the infection. Seven effectors were found to be novel and were examined in detail. All of the seven cell death effectors considered in the present study had no signal peptide despite being predicted to be potential cytoplasmic effectors (WolfPSORT). However, the effectors’ NN scores exceeded the threshold of 0.5 (SecretomeP 2.0), suggesting that they could be secreted through an unconventional system. Many studies have documented the non-conventional secretion of the effectors from fungi through extracellular vesicles, autophagosomes, or exocytosis [48,49]. Cysteine richness determines the structural stability of an effector in the extracellular environment following pathogen secretion. However, the effectors secreted to the host cytoplasm are low in cysteine [50]. For example, the hemibiotroph *Leptosphaeria maculans* secretes AvrLm1, an avirulence protein with only one cysteine residue [51]. Among the seven effectors, RS107_6 had a relatively high cysteine content (7), while RS107_4 had no cysteine residues. Therefore, it is reasonable to speculate that proteins with low cysteine content (<4) and without a signal peptide can also function as effectors.

Transcriptional induction of the effectors in phytopathogenic fungi are common during infection. Our results display that most of the effector proteins showed a significant upregulation during the course of infection, indicating their role as pathogenicity factors. In particular, the expression of the ubiquitin-like autophagy protein ATG12 (RS107_2) and Ribosomal_S8 protein (RS107_3) increased considerably by eight- and nine-fold, respectively. RS107_3 is a structural protein of the 40S subunit with a conserved ribosomal S8 domain; these proteins have been linked to haustoria development and sporulation in the stripe rust fungus, *Puccinia striiformis* f. sp. *tritici* [52]. The ribosomal proteins in phytopathogenic fungi have been found to be highly expressed during the early stages of disease development, most likely as a result of increased metabolic and translation activity [53]. However, the mechanism by which the ribosomal proteins contribute to fungal virulence will be the focus of future research. Autophagy is crucial for recycling the cytoplasmic constituents and cell survival in eukaryotes. In several fungal pathogens, including *M. oryzae*, *Verticillium dahliae*, and *Fusarium oxysporum,* autophagy is associated with fungal development and virulence [54,55,56]. These pathogens produce specialized infection structures to penetrate and enter the host cells; the recycling of intracellular components facilitated by autophagy helps to meet the demands of the nutrient and cytoplasmic material flow during the process of invasion. Thus, our in planta expression studies in rice demonstrated a significant upregulation of RS107_2, highlighting autophagy’s crucial role in the infection process.

Rab GTPase plays an important role in various cellular processes, including lipid metabolism, vesicular transport, protein secretion, and autophagy. In recent years, Rab GTPases have also been implicated in the virulence of several pathogenic fungi. For instance, disruption of the Rab8 homologue Bcsas1 in *Botrytis cinerea* resulted in small, compact colonies and reduced sporulation [57]. The rice blast fungus *M. oryzae* requires the Rab protein, MoRab5, for proper conidiation and appressorium formation, which are critical for its pathogenicity [58]. The higher expression of RS107_1 (Rab_18) during an infection implies the role of Rab_18 in fungal development and virulence through its vesicle trafficking and protein secretion activity.

Heat shock proteins (HSP)/chaperones are primarily involved in protein folding, unfolding, and translocation between compartments. HSPs are expressed constitutively performing housekeeping functions. Under stress, the expression of the HSPs is increased drastically, indicating its key role in cell survival. The pathogen-derived HSPs inducing strong immune responses in the host were reported in bacteria, protozoa, fungi, and nematodes [59]. In planta expression studies in rice revealed a reduced expression of RS107_5 (Cpn10) relative to mycelia, which is intriguing to hypothesize that the increased accumulation of RS107_5 transcripts could be perceived as potential threat signals, resulting in the immune response. Therefore, the reduced expression might favor the entry of pathogens while avoiding activation of the host defense. The effectors RS107_2, RS107_3, and RS107_6 had a significant upregulation and hence were found to be essential during the necrotrophic phase in *R. solani* infection.

In this study, we described the expression and purification of RS107_6 (metacaspase) from *R. solani*. The purified protein corresponded to ~36.5 kDa in size, which was also evident from the theoretical molecular weight (33.12 kDa) predicted using the ExPasy Protparam tool. The hexahistidine tag and the linker region derived from the pET-28a(+) expression vector accounted for the additional molecular mass observed. The molecular weight of RS107_6 was comparable to that of the metacaspases from various fungal species including *M. oryzae* MoMca1 (47 kDa) and MoMca2 (45 kDa), *Ustilago maydis* Mca1 (50 kDa), *S. cerevisiae* Yca1 (50 kDa), *Candida glabrata* CgMca-1 (48 kDa), *Schizophyllum commune*, and *Podospora anserina* PaMCA (77 kDa) [60,61,62,63,64,65]. Metacaspases are the cysteine proteases with calcium-dependent peptidase activity, principally involved in programmed cell death (PCD) and other non-apoptotic processes. Recent studies in *M. oryzae* and *U. maydis* have demonstrated the significance of metacaspases in the transition between developmental stages, such as hyphal growth, conidia germination, and appressorial morphogenesis, indicating their significance during vegetative growth. They were also found to be dispensable for the complete virulence of the fungi as the mutants resulted in a reduced disease severity [60,61]. Furthermore, metacaspases have been shown to play a crucial role in regulating cell death during plant–fungal interactions [53]. Therefore, the increased transcript abundance of RS107_6 at 72 hpi (necrotic phase) suggests the crucial role of apoptosis during sheath blight pathogen infection. Plant metacaspases also play an important role in regulating apoptotic cell death similar to pathogen metacaspases. For example, AtMC1 in *Arabidopsis thaliana* is reported to positively regulate HR-like cell death in response to the *Pseudomonas syringae* pv. *tomato* and *Hyaloperonospora arabidopsidis* infection [66], implying that the pathogenic fungi might hijack the plant metacaspase-mediated cell death pathway for its own benefit. Further studies are underway to assess the effect of expressed protein on symptom production and expression in the defense responsive genes in plants.

## 5. Conclusions

This study represents the first case of the characterization of seven novel cell death effectors (RS107_1 to RS107_7) in *R. solani*, a causal agent of rice sheath blight disease whose expression was found to be transcriptionally induced during infection, signifying the role of these effectors in disease development. The RS107_6 gene encoding for metacaspase has been found to persist, closely associated with intracellular protein aggregates generated during an event of stress exposure to the fungus. Identifying the rice target proteins involved in regulating the programmed cell death mechanism has provided insights into the defense pathway modulated by these effectors. Overall, our study provides the basis for further elucidation of the specific functions associated with each effector in *R. solani* and the precise molecular mechanism by which they regulate fungal development and virulence.

## Figures and Tables

**Table 1:** Primary sequence analysis and subcellular localization of cell death effectors computed using the ExPasy Protparam tool.

Cell Death Effectors	Conserved Domain	Subcellular Localization	Complete Coding Sequence (bp)	Amino Acid Residues	Molecular Weight (kDa)	Cysteine Content	Isoelectric Point(pI)	Instability Index	Aliphatic Index	GRAVY Index
RS107_1	Rab_18 domain	Cytoplasmic	642	213	23.15	3	5.41	27.99	78.17	-0.268
RS107_2	UbI-like ATG12 domain	Cytoplasmic-nuclear	423	140	15.75	2	9.23	34.36	81.57	-0.064
RS107_3	Ribosomal_S8	Cytoplasmic/apoplastic	393	130	14.66	1	10.33	37.32	113.77	-0.071
RS107_4	RRM_eIF3G	Cytoplasmic-nuclear	396	131	14.39	0	6.60	38.97	49.92	-0.886
RS107_5	Cpn10 domain	Cytoplasmic	297	98	10.12	1	9.8	32.07	98.47	0.118
RS107_6	Peptidase_C14	Cytoplasmic	906	301	33.10	7	5.39	44.73	75.22	-0.437
RS107_7	GAPDH	Cytoplasmic	1032	343	36.85	2	6.34	21.79	90.35	-0.103

**Table 2:** Role of rice target genes identified using molecular docking studies in defense response.

Rice Target Genes	Protein Name	Role in Plants	References
LOC_Os04g56480 (OsPELOTA)	PELOTA protein	mRNA surveillance and quality control; enhance resistance against rice bacterial blight pathogen by activating salicylic acid pathway	Kong et al. [37]; Zhang et al. [38]
LOC_Os01g56330 (FLR2)	Feronia protein	Receptor-like kinases; recognize the PAMPs and trigger oxidative bursts	Yang et al. [39]
LOC_Os03g10750 (SPL35)	ASC-1 complex subunit	Ribosome-associated quality control; regulates cell death and defense response through its ubiquitination and vesicular trafficking pathways	Juszkiewicz et al. [40]; Ma et al. [41]
LOC_Os01g02720 (SPL33)	HBS-1-like protein	mRNA surveillance and quality control	Kong et al. [37]
LOC_Os09g38580 (OsCNGC9)	Cyclic nucleotide-gated ion channel protein	Passages for conducting calcium ions into the cytosol in response to pathogen invasion as an early event in defense signaling	Nawaz et al. [42]
LOC_Os03g06410 (OsEDR1)	Serine–threonine protein kinases/mitogen- activated protein kinases	Signaling and plant defense	Afzal et al. [43]; Shen et al. [44]
LOC_Os02g36974 (OsGF14e)	14-3-3-like protein GF14E	Negatively regulates the induction of plant defense response genes, cell death, and contributes to broad-spectrum resistance in rice	Manosalva et al. [45]

**Table 3:** Purification summary of recombinant RS107_6 protein in *R. solani* using Ni-NTA affinity column chromatography.

Purification Step	Protein Concentration (mg/mL)	Total Protein (mg)	Yield (%)	Purification (Fold)
Crude lysate	1.85	27.7	100	1
Affinity purification	1.16	11.6	41.8	0.4

## Data Availability

All genetic data are available from the GenBank repository and Accession number of the genes are provided in the manuscript and supplementary data.

## Supplementary Materials

The following supporting information can be downloaded at: https://www.mdpi.com/article/10.3390/microorganisms11040920/s1, Table S1. List of primers with restriction sites added to the 5′ ends of the sequences used for PCR-amplification and cloning of the cell death effectors. Table S2. List of primers used in gene expression studies of cell death effectors. Table S3. Accession numbers obtained for the identified cell death effectors RS107_1 to RS107_7. Figure S1. Phylogenetic analysis of ITS rDNA sequence of RS107 with its reference sequences using the Neighbor-joining algorithm in MEGA-11. Figure S2. Phylogenetic tree constructed from aligned amino acid sequences of (A) RS107_1 (B) RS107_2 (C) RS107_3 (D) RS107_4 (E) RS107_5 and (F) RS107_7 with their reference proteins using the Neighbor-joining algorithm in MEGA11. Figure S3. Three-dimensional modelling and validation of cell death effectors (A) 3D structures of effectors RS107_1 to RS107_7 predicted using RaptorX server. (B) Validation of developed models by computing RC plot statistics. Distribution of amino acid residues in most favored region (red), additionally allowed region (bright yellow), generously allowed region (yellow) and disallowed region (white) is indicated. Figure S4. Three-dimensional modelling and validation of rice target genes (A) 3D structures of rice interacting partners predicted using RaptorX server. (B) Validation of developed models by computing RC plot statistics. Distribution of amino acid residues in most favored region (red), additionally allowed region (bright yellow), generously allowed region (yellow) and disallowed region (white) is indicated.
