# Supplementary material for: Insights on Novel Effectors and Characterization of Metacaspase (RS107_6) as a Potential Cell Death-Inducing Protein in Rhizoctonia solani
Source: Microorganisms. 2023 Apr 1;11(4):920. doi: 10.3390/microorganisms11040920 (PMC10143347; doi:10.3390/microorganisms11040920)
Supplement: Supplementary file 1 [file microorganisms-11-00920-s001.zip › microorganisms-2304706-supplementary.pdf]

SUPPLEMENTARY FILE

Table S1. List of primers with restriction sites added to the 5' ends of the sequences used for PCR-amplification and cloning of the cell death effectors.

**Effector**	**Primers**	**Sequence (5'-3')**	**Product length (bp)**
**RS107_1**	*F*	ATGAACGATACAGGAAACTCG	642
	R	CTAGCAGGCACATCCTCC	
**RS107_2**	*F*	ATGTCCAATCCTCTAACAGACGG	423
	R	TTAGAACGGTCTAGGCTGCTGAGG	
**RS107_3**	*F*	ATGGTTCGCGTCTCTGTCTT	393
	R	TTAGTACACATATCCAAGGAGC	
**RS107_4**	*F*	ATGCCTCCACCGGAGGAT	396
	R	CTATCCAGCCCTGGGTTCT	
**RS107_5**	*F*	ATGGTATGTCCCGCGGCCT	297
	R	TCAAGCGCTTACGTCTTCGCC	
**RS107_6**	*F*	ATGTGTACCGGAAAGAAAAAAG	906
	R	TCAAGCGATGAACAAGATG	
**RS107_6 with restriction site**	*BamHI F*	CGC*GGATCC*ATGTGTACCGGAAAGAAAAAAG	906
	HindIII R	GGG*AAGCTT*TCAAGCGATGAACAAGATG	
**RS107_7**	*F*	ATGACAGTCAAAGTTGG	1032
	R	TCAGGCGGCTTCTGCGT	

Table S2. List of primers used in gene expression studies of cell death effectors.

Effector	Sequence (5'-3')
**RS107_1F**	TACGACAGAGGAAGGTAAGG
**RS107_1R**	GCCCACACGTGTACTATTT
**RS107_2F**	TCTCCAAAGTCGTGGTGCG
**RS107_2R**	AGGAAATGAGCACCGACCAG
**RS107_3F**	GAAAGCGTCAGGTCCTTATC
**RS107_3R**	TTGAAGCGAGGAGAGATGA
**RS107_4F**	TACGTTCCTCCCTCTATGC
**RS107_4R**	GATCCCGACCAACGTAAAC
**RS107_5F**	GTCTGTCGTCCCTCTCTT
**RS107_5R**	TCTCCCTTCTTGTTCCGT
**RS107_6F**	CAAAGACGCTCACCCTAATG
**RS107_6R**	GCCCTGCATGTTCAAAGT
**RS107_7F**	TGCACACATCAAGGAGAAC
**RS107_7R**	TGCACCGTAGTCATCAAAC

Table S3. Accession numbers obtained for the identified cell death effectors RS107_1 to RS107_7.

Gene name/Effector	Accession number
RS107_1	OQ389682
RS107_2	OQ389683
RS107_3	OQ389684
RS107_4	OP889287
RS107_5	OQ389685

RS107_6	OP889286
RS107_7	OQ389686

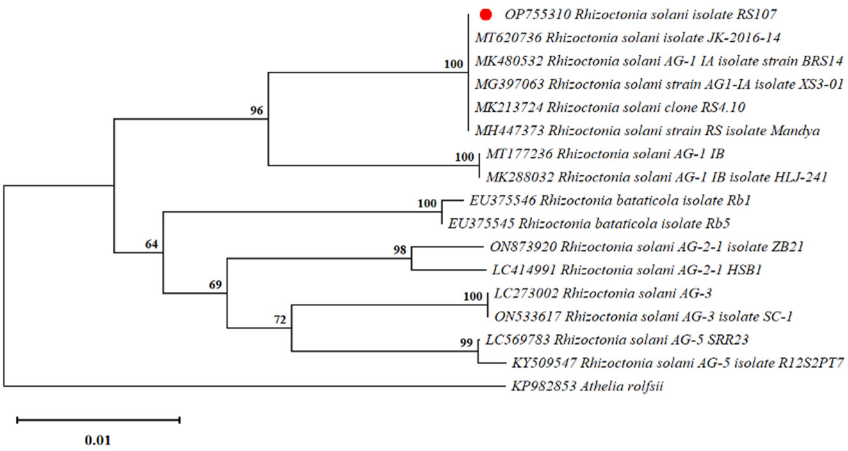

Figure S1. Phylogenetic analysis of ITS rDNA sequence of RS107 with its reference sequences using the Neighbor-joining algorithm in MEGA-11.

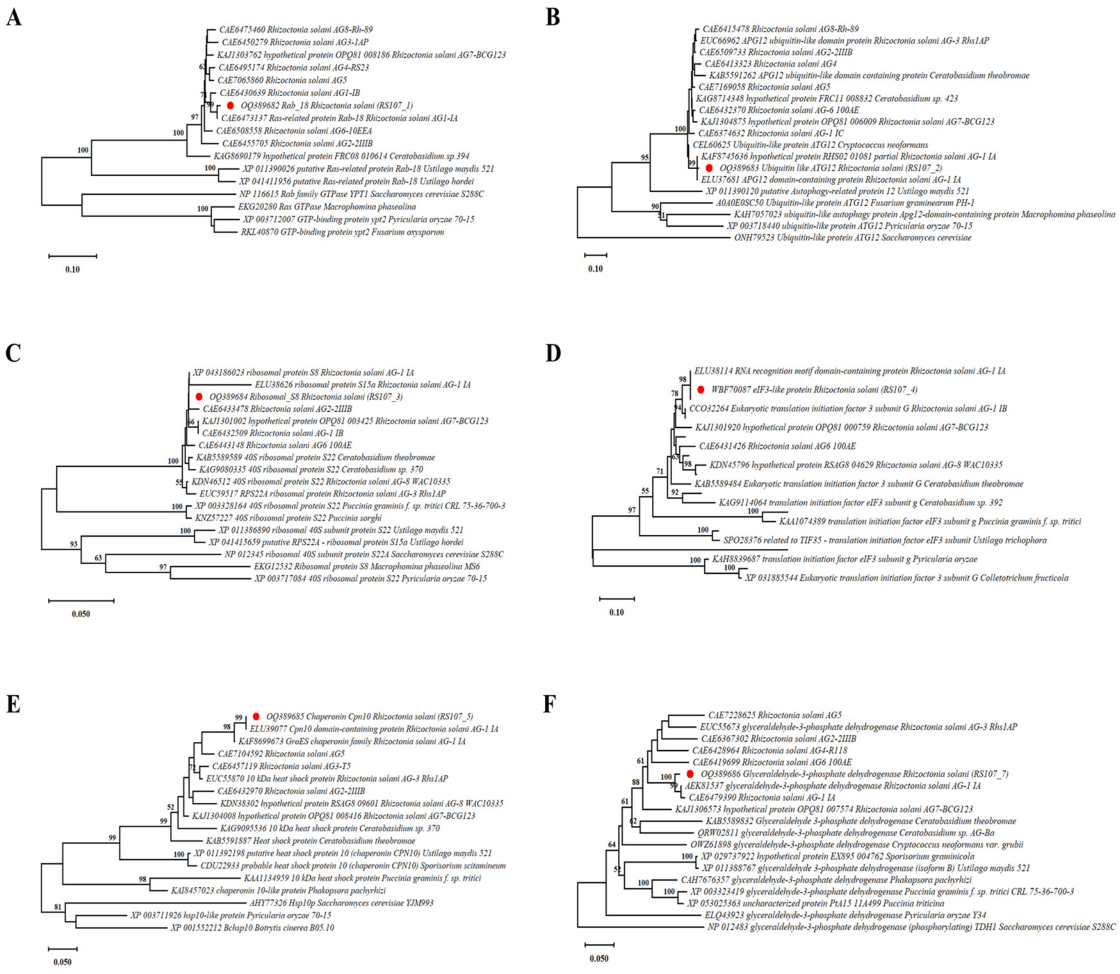

Figure S2. Phylogenetic tree constructed from aligned amino acid sequences of **(A)** RS107_1 **(B)** RS107_2 **(C)** RS107_3 **(D)** RS107_4 **(E)** RS107_5 and **(F)** RS107_7 with their reference proteins using the Neighbor-joining algorithm in MEGA11.

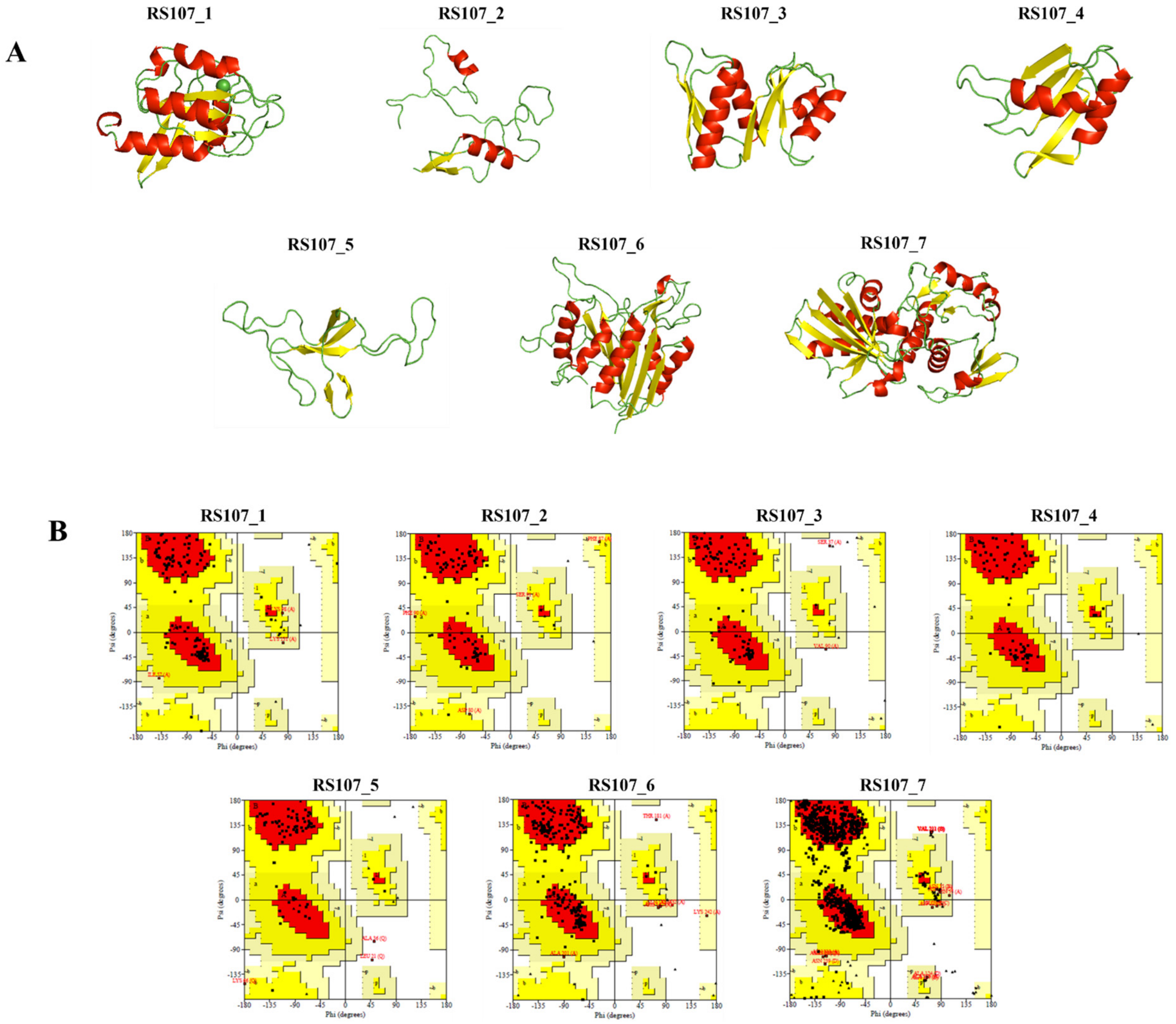

Figure S3. Three-dimensional modelling and validation of cell death effectors **(A)** 3D structures of effectors RS107_1 to RS107_7 predicted using RaptorX server. **(B)** Validation of developed models by computing RC plot statistics. Distribution of amino acid residues in most favored region (red), additionally allowed region (bright yellow), generously allowed region (yellow) and disallowed region (white) is indicated.

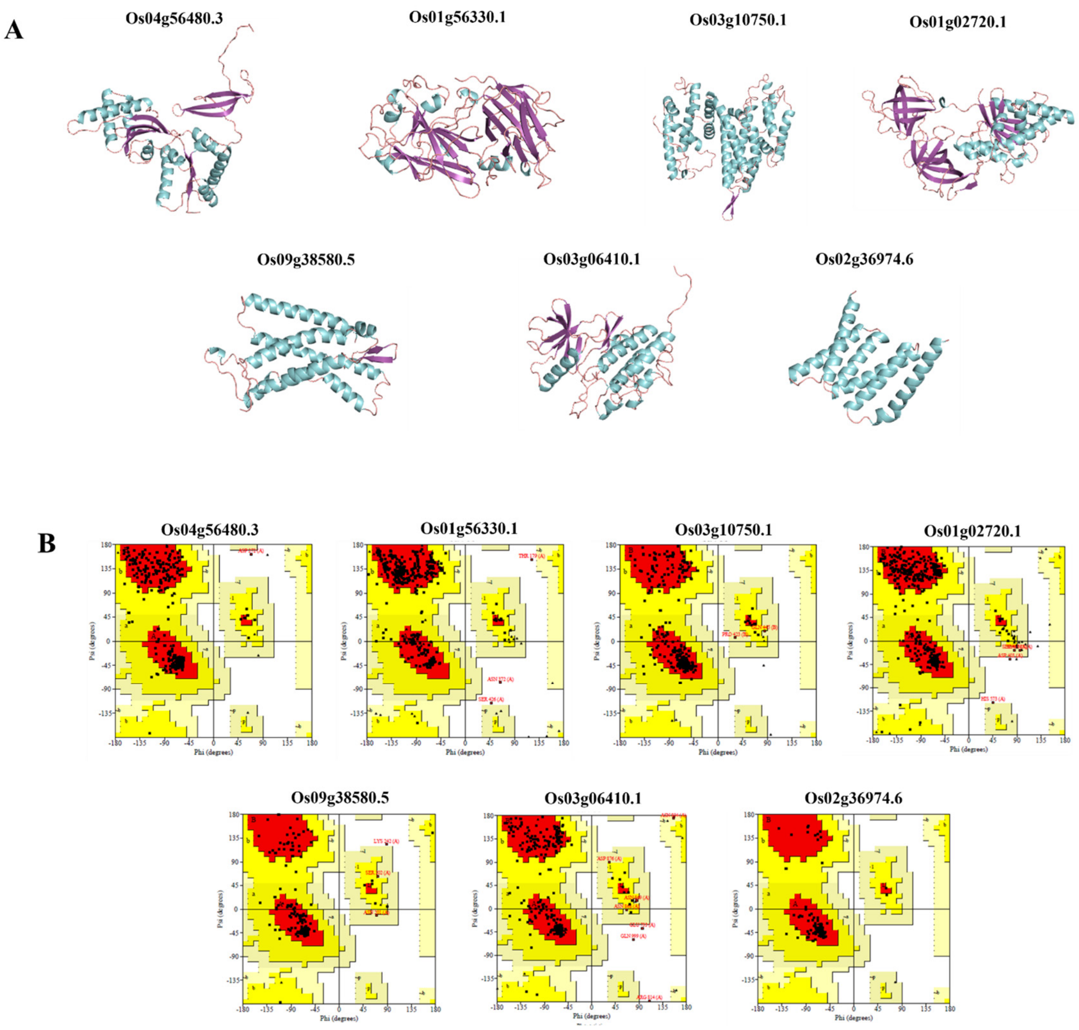

Figure S4. Three-dimensional modelling and validation of rice target genes **(A)** 3D structures of rice interacting partners predicted using RaptorX server. **(B)** Validation of developed models by computing RC plot statistics. Distribution of amino acid residues in most favored region (red), additionally allowed region (bright yellow), generously allowed region (yellow) and disallowed region (white) is indicated.
